# Periodontal Disease - A Late Complication of Head and Neck Cancer Radiotherapy

**DOI:** 10.1177/10732748241255845

**Published:** 2024-05-17

**Authors:** Ella Brandt, Mutlu Keskin, Ismo T. Räisänen, Antti Mäkitie, Tommi Pätilä, Timo Sorsa, Shipra Gupta

**Affiliations:** 1 Department of Oral and Maxillofacial Diseases, 560027 University of Helsinki and Helsinki University Hospital, Helsinki, Finland; 2 Oral and Dental Health Department, Altinbas University, Istanbul, Turkey; 3 Department of Otorhinolaryngology- Head and Neck Surgery, 560027 Helsinki University Hospital and University of Helsinki, Helsinki, Finland; 4 Research Program in Systems Oncology, Faculty of Medicine, 560027 University of Helsinki, Helsinki, Finland; 5 Division of Pediatric Surgery, Children’s Hospital, 560027 Helsinki University Hospital and University of Helsinki, Helsinki, Finland; 6 Division of Periodontology, Department of Dental Medicine, Karolinska Institutet, Huddinge, Sweden; 7 Oral Health Sciences Centre, 29751 Post Graduate Institute of Medical Education & Research, Chandigarh, India

**Keywords:** matrix metalloproteinases, mucositis, radiation, cancer, oral health, xerostomia, antimicrobial, radiotherapy, biomarkers, diagnostics, oral hygiene

## Letter to the Editor

We read with great interest the original paper recently published by Austin J. Lovoli entitled “Severe oral mucositis after intensity-modulated radiation therapy for head and neck cancer”. The authors discuss how by the end of definitive or adjuvant intensity-modulated radiotherapy (IMRT) for primary head and neck cancers (HNC), 568 (98.6%) out of a total of 702 patients developed at least some degree of oral mucositis while 360 patients (62.5%) developed severe oral mucositis.^1^ The authors also discussed how inflammation and ulcers characteristic of oral mucositis negatively affect the patient’s quality of life by reducing their ability to properly masticate their food, causing xerostomia, dysphagia, and hampering their speech.^1^

Here, it is noteworthy to mention that not only mucositis but also periodontitis and its potential escalation are common following radiotherapy. When the jaws are in the field of radiation, around 70% of patients suffer from increased periodontal attachment loss after completion of radiotherapy.^2^

### Periodontal Disease- As Late Complication of Head and Neck Cancer Radiotherapy

Golub et al. (2006) introduced a ‘two-hit’ model to explain the connection between radiation-induced oral mucositis and periodontitis.^3^ In this model, they propose that the initial ‘hit’ is inflammation at the periodontium level, known as periodontitis. This is followed by a second ‘hit,’ which is exposure to radiation. According to the model, this sequential occurrence can result in an exaggerated host response, manifesting as oral mucositis. Notably, the model also suggests a reciprocal relationship, where radiation-induced oral mucositis can act as the first ‘hit,’ exacerbating the inflammatory response in the development of periodontitis, which serves as the second ‘hit’ in this context.^3^

If this hypothesis is valid, as suggested by Khaw et al. (2013), it becomes logical to address both ‘hits' simultaneously to achieve optimal benefits for conditions like radiation-induced oral mucositis and periodontal degeneration.^4^ In other words, targeting both the initial periodontitis and the subsequent radiation exposure could offer a more effective strategy for managing and preventing the exacerbation of these oral health conditions.

Periodontitis, hence, has been considered a late complication of radiotherapy.^5^ Oral dysbiosis following chemotherapy and the hypoxic and hypovascular changes following radiotherapy definitely have an adverse effect on periodontal health, aggravating pre-existing periodontitis by balancing host immune defense and oral microbiome.

An irradiated periodontium, with its decreased repair ability, is more vulnerable to infection, possibly inducing osteoradionecrosis, a feared late complication of radiotherapy.

Marques and Dib (2004) reported loss of attachment in 70.3% of their patients following radiation therapy, highlighting the need to evaluate periodontal status before and following radiation therapy to help ensure that periodontal health is maintained in these patients.^5^

They concluded that the local and systemic susceptibility of the host following radiation, along with oral dysbiotic periodontopathobionts are responsible for the periodontal degeneration.

Ensuing gingival recession, decreased opening of the jaws, and xerostomia further reduce the ability of the patient to maintain oral hygiene, worsening the periodontal health.

Patients with severe periodontal disease have been found to be more prone to developing osteoradionecrosis, especially if aggressive periodontal treatment has not been performed to achieve an oral foci free state at least 10-15 days prior to starting radiotherapy.^6^

It then becomes mandatory to control plaque accumulation and biofilm composition to prevent the colonization of periodontal pockets by pathogens in these patients with compromised local defence.

To keep a tight check on the periodontal health of an individual indicated for or undergoing radiotherapy, commercially available, simple, non-invasive, and easy-to-use active MMP-8 point-of-care-test kits are a viable option.

### Role of Active MMP-8 in Oral Mucositis and Related Periodontal Disease

As previously published by Brandt et al. (2023), side effects following radiotherapy can be attributed to a change in the neutrophil/lymphocyte ratio, which is also a dependable indicator of subclinical inflammation, both at systemic and periodontal levels.^7^ Matrix metalloproteinase (MMP)-8 is a known neutrophil-sourced collagenase or collagenase-2, and is also the major collagenolytic protease activated in inflamed periodontium and oral fluids.^7^

Al-Dasooqi et al. (2010), in their work on animal models of mucositis, found MMPs to be up-regulated. They concluded that MMPs play a key role in tissue injury and inflammation.^8^ Using the same rodent model, they also reported a significant alteration in both gene expression and tissue levels of MMPs and tissue inhibitor of metalloproteinase (TIMPs) following cytotoxic chemotherapy.^8^ The augmentation in the expression profiles of MMPs and their inhibitors correlated with histopathological alterations observed in the tissue following chemotherapy.^4^

Brandt et al. (2023) reported an increase in the aMMP-8 levels in mouthrinse after three weeks of radiotherapy, proving how the treatment triggered or induced the neutrophils to degranulate, activating MMP-8. aMMP-8 levels predictably correlate with periodontal status.^7^ A significant positive correlation between aMMP-8 levels (both pre- and post-radiotherapy) and the difference between the mean of probing depths pre-and post-radiotherapy have also been established previously.^9^

**Figure 1.**
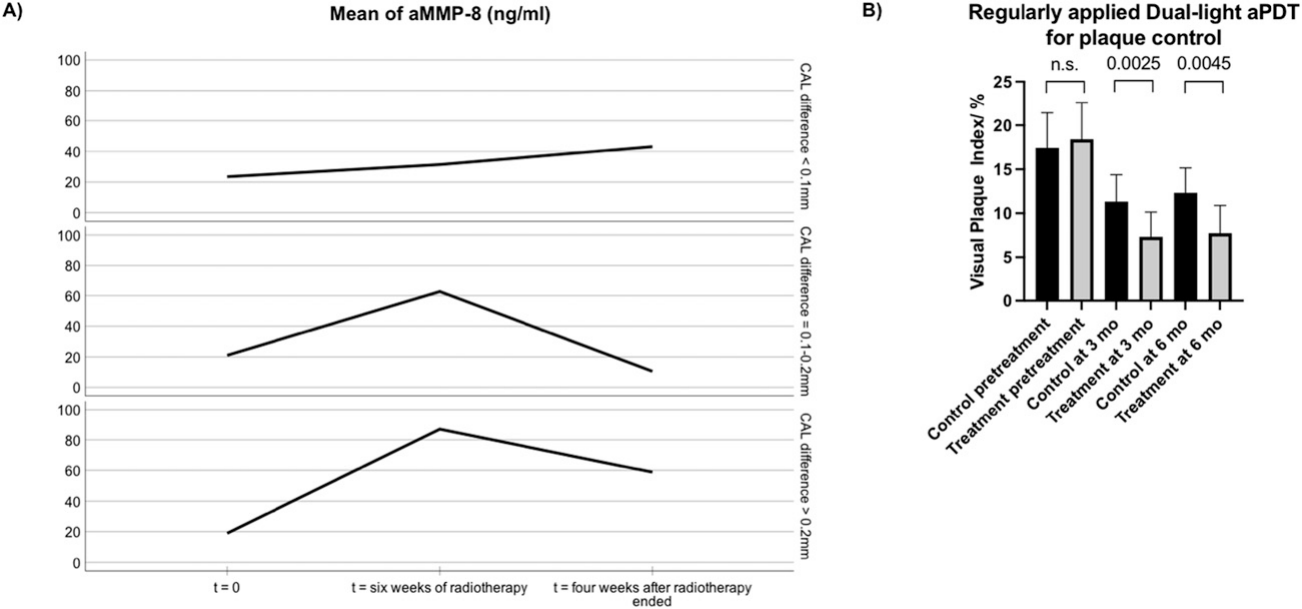
(A) Mean levels of aMMP-8 (ng/ml) among 21 HNC-patients (as described in Brandt et al. (2023)) at t = 0 weeks, after 6 week radiotherapy period (t = 6 weeks) and 4 weeks after radiotherapy treatment had ended (t = 10 weeks); grouped by CAL difference between pre-radiotherapy (t = 0 weeks) and 4 weeks after the radiotherapy period (t = 10 weeks) (groups of CAL below 0.1 mm [N = 10], between .1 and 0.2 mm [N = 5] and over 0.2 mm [N = 6]. (B) Daily applied aPDT significantly reduced the amount of plaque in periodontitis patients compared to controls. Both groups received SRP and standard oral hygiene instructions. The number of patients was 51 in the treatment group and 49 in the control group. Visual plaque index is expressed in median (SD), and the Mann-Whitney test was used for analysis.

Figure 1(A) depicts an association between aMMP-8 levels and clinical attachment loss (CAL) following 6 weeks of radiotherapy (N = 21): Higher the average increase in aMMP-8 levels, higher was the recorded CAL loss.

Hence, simple and accurate kits commercially available in the market to measure aMMP-8 can be utilized for its measurement to help evaluate the risk of periodontal changes following radiotherapy. They are accurate and have a high sensitivity and sensitivity, leading to real-time periodontal status evaluation. The tests are not sensitive to technique and can be easily used by patients or non-experts at home or in medical or dental specialist offices.^10^

Ultimately, the paramount factor in periodontal treatment lies in the effectiveness of regular oral care practices carried out at home.

### Home-Applied Antibacterial Photodynamic Therapy in Dental Plaque Control

Radiotherapy often leads to xerostomia associated with decreased salivary pH and buffering capacity, as well as changes in the oral microbiome including cariogenic and periodontopathogenic bacteria and candida species and these dysbiotic changes can escalate and promote mucositis and related developing periodontitis in the patients with reduced immune defence due to radiotherapy.^11-13^ Also both radiation induced oral mucositis and periodontitis are linked by the presence of systemic inflammation, as proposed by the ‘two hit’ model hence the healing response in the patients having mucositis lesions will be affected considerably by host inflammatory reaction, and in some individuals with an underlying dysregulation of the inflammatory response (i.e. co-existing periodontitis and mucositis).^4^

Even the best toothbrushes remove only about 65% of dental plaque. Hence, a need arises for adjuvants in periodontal treatment. Because of the inherent problems related to the existing means, like antibiotics and chlorhexidine, the optimal adjuvant method for improved plaque control is yet to be found. Recently, due to the rapid development in the LED technology, antibacterial photodynamic therapy (aPDT) and antibacterial blue light (aBL) have emerged as predictable solutions for oral home care. Benefits of light induced antibacterial treatments are high efficacy, local action targeted on plaque, no resistance formation and ease-of-use.

Dual-light therapy, where aPDT and aBL are applied simultaneously, has shown significant antibacterial efficacy in biofilms and reduced dental plaque development.^14-17^ The antimicrobial effect of the combined use of aPDT and aBL is based on the principle that visible light activates an externally applied photosensitizer (in the case of aPDT) or internal molecules in bacteria, such as porphyrins and flavins (photosensitizers in the case of aBL), producing reactive oxygen species (ROS) that kill bacteria indiscriminately through oxidative bursts. A repetitively applied combination of aBL and aPDT has been shown to significantly increase bactericidal action, especially on matured biofilms.

Lumoral^®^ is a novel CE-marked medical device in a mouthguard form, designed for use at home by the patient himself, enhancing compliance. Following one minute of rinsing with Indocyanine green mouth rinse provided with the device, a 50:50 combination of 405 nm aBL and 810 nm aPDT light is passed through the device to activate its antibacterial effect. Selective adherence of ICG to dental plaque bacteria leads to targeted antibacterial activity.

Regular dual-light treatment by Lumoral^®^ has been found to reduce inflammation and proinflammatory markers including aMMP-8, in peri-implant disease and has shown benefits in maintaining oral hygiene at home. Figure 1(B) presents how daily applied aPDT significantly reduced the amount of plaque in periodontitis patients (N = 51) compared to controls (N = 49). Both groups received SRP and standard oral hygiene instructions.

## Conclusion

Periodontal disease evaluation both before and following radiotherapy is essential for preserving the oral-health-related quality of life in oncology patients. Hence, it is an obligation both on the part of the oncologist and the dentist to emphasize the importance of good oral hygiene in patients undergoing radiation therapy. Non-invasive biomarkers like aMMP-8 and the POCT are available for periodic screening of periodontal disease status. Antibacterial photodynamic therapy, when performed regularly at home, helps keep the oral microbiome in check in cancer survivors. We suggest that with aMMP-8 POCT diagnostics and regularly applied aPDT-treatment modality, one can eventually prevent, diagnose, treat, and control the radiotherapy patients’ oral and periodontal health more effectively.

## Data Availability

The data that support the findings of this study are available on reasonable request from the corresponding author.*
